# Regional mudstone compaction trends in the Vienna Basin: top seal assessment and implications for uplift history

**DOI:** 10.1007/s00531-023-02331-4

**Published:** 2023-07-28

**Authors:** Lukas Skerbisch, David Misch, Michael Drews, Harald Stollhofen, Reinhard F. Sachsenhofer, Klaus Arnberger, Volker Schuller, Andras Zamolyi

**Affiliations:** 1 Montanuniversitaet Leoben, Peter Tunner Straße 5, 8700 Leoben, Austria; 2 Technical University of Munich, Munich, Germany; 3 Friedrich-Alexander University (FAU) Erlangen-Nuremberg, Erlangen, Germany; 4 OMV Exploration and Production GmbH, Gänserndorf, Austria

**Keywords:** Vienna Basin, Mudstone compaction, Top seal quality, MICP, BIB-SEM, Helium pycnometry, Uplift history

## Abstract

Seal quality assessment is not only essential in petroleum systems studies but also in the context of other geo energy applications such as underground hydrogen storage. Capillary breakthrough pressure controls top seal capacity in the absence of faults or other discontinuities. In basins that lack measured capillary pressure data (e.g., from drill cores), regional compaction-porosity trends can be used as a first prediction tool to estimate the capillary properties of mudstones. Mathematical compaction models exist but need to be calibrated for each basin. This study aims to establish a compaction trend based on theoretical models, then compare it with theoretical maximum hydrocarbon column heights inferred from true measured capillary pressure curves. Middle to upper Miocene mudstone core samples from the Vienna Basin, covering a broad depth interval from 700 to 3400 m, were investigated by X-ray diffractometry, with an Eltra C/S analyzer, and by Rock–Eval pyrolysis for bulk mineralogy, total organic carbon, and free hydrocarbon contents. Broad ion beam—scanning electron microscopy, mercury intrusion capillary porosimetry, and helium pycnometry were applied to obtain pore structural properties to compare the mathematical compaction models with actual porosity data from the Vienna Basin. Clear decreasing porosity depth trends imply that mechanical compaction was rather uniform in the central Vienna Basin. Comparing the Vienna Basin trend to global mudstone compaction trends, regional uplift causing erosion of up to ~ 500 m upper Miocene strata is inferred. A trend of increasing Rock–Eval parameters S1 and production index [PI = S1/(S1 + S2)] with decreasing capillary sealing capacity of the investigated mudstones possibly indicates vertical hydrocarbon migration through the low-permeable mudstone horizons. This observation must be considered in future top-seal studies for secondary storage applications in the Vienna Basin.

**Supplementary Information:**

The online version contains supplementary material available at 10.1007/s00531-023-02331-4.

## Introduction

**Fig. 1:**
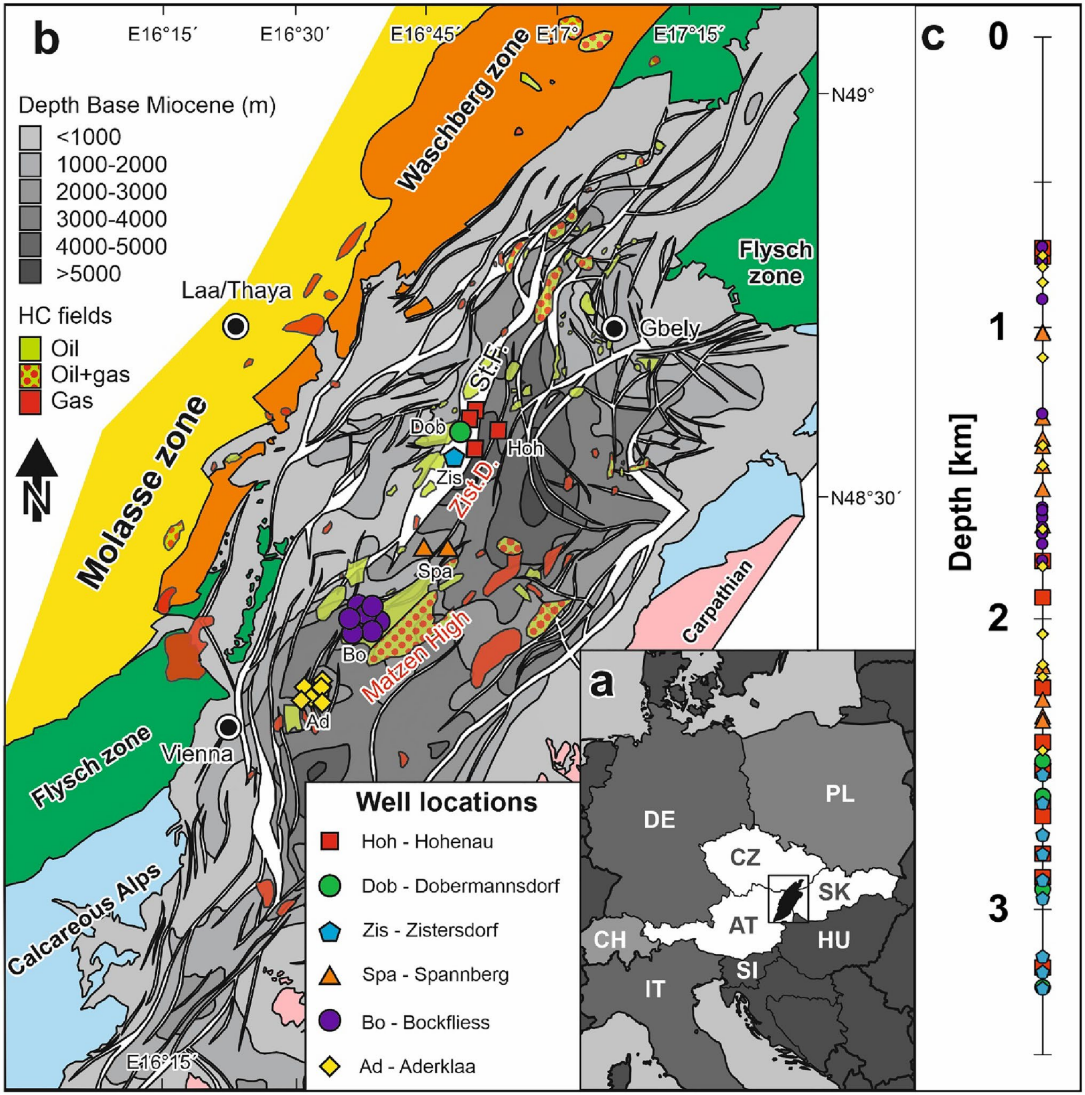
**a** Location of the Vienna Basin in Central Europe; black rectangle refers to (**b**). **b** Positions of sampled wells in the Vienna Basin in a combined structural and regional geological map [modified from Rupprecht et al. (2018)]. **c** Investigated core samples (68) from the Vienna Basin aligned according to their depth. Note that the samples are colour coded according to their well positions shown in (**b**). HC-fields hydrocarbon fields, St.F. Steinberg Fault

The Vienna Basin extends from Austria to Slovakia and the Czech Republic (Fig. 1a). The Miocene basin fill reaches a maximum thickness of more than 5 km (Fig. 1b) and overlies various basement units, which include from base to top crystalline basement, autochthonous Mesozoic sediments, Cenozoic foreland basin deposits, and the Alpine nappe system (Flysch Zone, Calcareous Alps; Wessely 1988; Arzmüller et al. 2006). Oil and gas have been detected in the Miocene (Ottnangian to Pannonian) basin fill and in basement units, making the Vienna Basin one of the major Central European hydrocarbon provinces. To date, 160 fields have been detected with cumulative recoverable reserves of approximately 1660 Mmboe (Boote et al. 2018).

Due to extensive hydrocarbon exploration, the structure and evolution of the Vienna Basin and its petroleum system are generally well understood (Wessely 1988; Arzmüller et al. 2006; Rupprecht et al. 2018). However, knowledge gaps exist regarding the young (post-Pannonian;  < 9 Ma) uplift history of the basin. While the distribution and quality of the source and reservoir rocks have been studied in detail, investigations of the quality of seal rocks have been largely neglected given the large number of apparently preserved hydrocarbon accumulations. Nevertheless, as a reliable migration history model of the basin may lead to new discoveries and a longer production lifespan, top seal quality became increasingly relevant as a success factor in recent years. Furthermore, top seal-related issues are of great relevance in the context of secondary storage safety (e.g., CH_4_, CO_2_, H_2_).

The parameter that theoretically defines the static top seal capacity of a presumably water-wet seal is the capillary entry or breakthrough pressure, as it limits the maximum amount of buoyant fluid that may be accommodated by a reservoir structure (Schowalter 1979). In such a purely top seal-controlled scenario (excluding other factors like trap geometry or charging), the density-driven buoyancy pressure of the entire fluid column plus optional additional hydrodynamic pressure components (e.g., due to overpressure of underlying formations) has to be counterbalanced by the capillary forces acting in the seal rock. Thus, an assessment, particularly of large structures, should always cover the calculation of a maximum fluid column height based on a threshold breakthrough pressure estimation as well.

In the absence of core material, the seal capacity of mudstones, which is essentially a function of pore throat distributions for a given fluid system, can be estimated based on assumed “Normal compaction trends”. Such porosity/depth relationships have been established for different basins and compaction mechanisms (Athy 1930; Hedberg 1936; Weller 1959; Meade 1966; Sclater and Christie 1980; Baldwin et al. 1985; Yang and Aplin 2004; Mondol et al. 2007). However, these normal compaction trends only provide a rough estimate and may be overly simplified to represent the situation at an individual basin scale. Many factors such as the pore pressure conditions, mineralogic composition, variations in grain size and fabric, or diagenetic processes strongly influence the compaction behaviour of the fine-grained sediments within a respective basin (Bjørlykke and Høeg 1997; Bjørlykke 1998, 1999, 2014; Mondol et al. 2007; Fawad et al. 2010; Drews et al. 2018). Hence, this work aims at testing the general suitability of theoretical compaction trend models for the prediction of actual petrophysical porosity and capillary pressure data in the Vienna Basin. For this purpose, a set of 68 Miocene (Pannonian, Sarmatian, and Badenian) seal rocks of the Vienna Basin, covering a broad depth interval from 700 to 3400 m (Fig. 1c), was investigated in detail by X-ray diffractometry, He-pycnometry, mercury intrusion capillary porosimetry, broad ion beam—scanning electron microscopy, as well as Rock–Eval pyrolysis. The comparison of a regional porosity—depth trend obtained from well-characterized mudstone intervals with modelled normal compaction trends should facilitate future calibrations of general compaction and resulting seal capacity models (Yang and Aplin 1998, 2004, 2007, 2010). Furthermore, the application of Rock–Eval pyrolysis for free hydrocarbon detection is introduced as a potential geochemical tool to reveal vertical hydrocarbon migration through low-permeable mudstones. Apart from the top seal assessment, the detected porosity—depth trend will also be used to shed light on the post-Pannonian erosion history of the basin.

## Geological setting

The sedimentary succession in the Vienna Basin area is subdivided into three tectonostratigraphic units (Wessely 1988). The autochthonous unit consists of Jurassic to Cretaceous syn- and postrift deposits and Paleogene molasse sediments. The allochthonous unit consists of the Alpine-Carpathian nappe complex (including Flysch Zone and Calcareous Alps), which overthrusted the autochthonous units during Late Oligocene and early Miocene time (Beidinger and Decker 2014).

The Miocene fill of the Vienna Basin forms the uppermost tectonostratigraphic unit. Miocene deposition commenced during an early Miocene (Ottnangian–Karpatian) piggy-back stage of basin evolution (Steininger et al. 1986; Seifert 1996; Wessely 2000). Following a tectonic phase, which caused strong tilting and erosion of lower Miocene sediments, sedimentation continued in the middle and late Miocene time in a basin controlled by pull-apart and extensional tectonics. Sediment accumulation rates were high and rather uniform during the middle Miocene time (0.9–1.1 m/kyr), but typically lower (0.4–0.9 m/kyr) during the late Miocene (Hölzel et al. 2008; Lee and Wagreich 2017; Harzhauser et al. 2019, 2022). The structure of the Miocene basin is dominated by two more than 5 km deep depressions separated by ‘Central Highs’ (e.g. Matzen High Fig. 1b). The depressions are bordered towards the west by major fault zones with vertical displacements of up to 6000 m (Steinberg fault; Fig. 1b). Faults bordering the depocentres towards the (south)east are less prominent. The architecture of the middle and upper Miocene sediments is controlled by five third-order sequences (Ba1 to Ba3; Sa1; Pa1), which can be linked to the global 3rd order sea-level cycles of Haq et al. (1988) and Hardenbol et al. (1999)

**Fig. 2:**
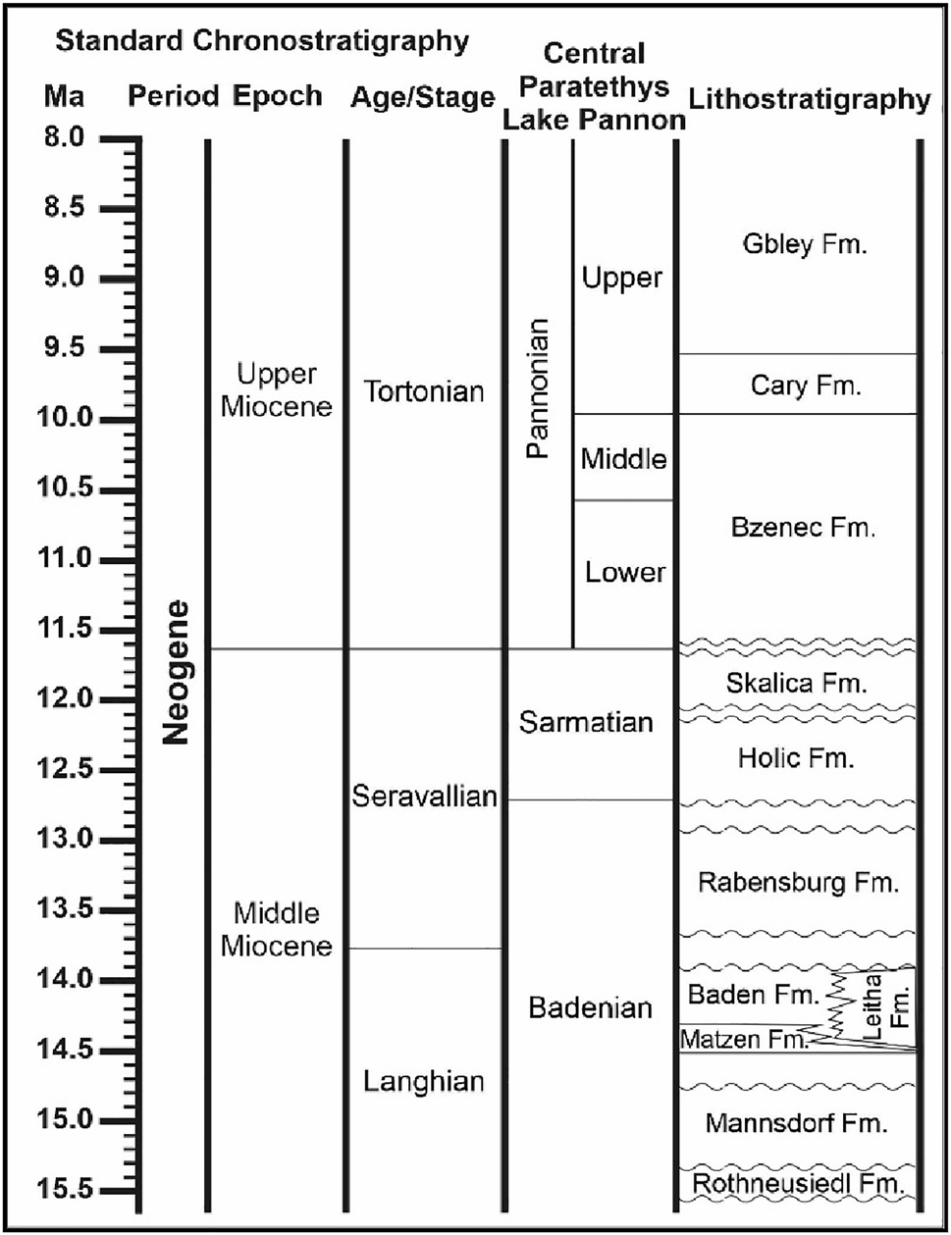
Stratigraphic chart relating the lithostratigraphic units with standard chronostratigraphic stages [after Harzhauser and Piller (2004a), Gradstein et al. (2020), Harzhauser et al. (2020)]

The Badenian (~ 16–12.7 Ma, Fig. 2) succession (Baden Group) includes three depositional cycles (Ba1 to Ba3) (Harzhauser et al. 2020; Siedl et al. 2020):

(i) Lower Badenian (Ba1) sediments include braided river deposits (Rothneusiedl Fm.) in the lower and marine delta sediments in the upper part (Mannsdorf Fm.);
(ii) The middle Badenian sequence Ba2 comprises transgressive sandstones in the lower part (Matzen Fm.) overlain by open marine clay and marl with silt and fine sand intercalations (Baden Fm.). In marginal areas, the Baden Formation interfingers with corallinacean limestones (Leitha Fm.; Riegl and Piller 2000). The maximum flooding surface of the Ba2 cycle represents the most extensive flooding event of the basin (Siedl et al. 2020). Water depth ranged around 250 m (Hohenegger et al. 2008; Kranner et al. 2021)
(iii) The upper Badenian sequence Ba3 follows after an erosional event caused by a sea level drop at the Langhian/Serravallian boundary (Piller et al. 2022). During the deposition of Ba3, the topographic highs were drowned and the last fully marine delta systems were deposited (Rabensburg Fm.; Harzhauser et al. 2020; Siedl et al. 2020). Thin anhydrite layers have been detected in the basal parts of the Rabensburg Fm. (Harzhauser et al. 2018).

The *Sarmatian* (~ 12.7–11.6 Ma, Fig. 2) succession is up to 1000 m thick and represents the third-order sequence Sa1 (Harzhauser and Piller 2004b). It includes the lower Holíc Formation, composed of grey calcareous claystones, siltstones, and rare acidic tuffite layers (Harzhauser and Piller 2004a) and the upper Skalica Formation. The latter contains marlstones and silt- to sandstones as well as conglomerates, incorporating mixed siliciclastic-calcareous deposits such as oolites, rock-forming coquinas, and foraminiferal bioconstructions (Eleéko and Vass 2001). The average water depth of the Sarmatian Sea was estimated at around 50 m (Kranner et al. 2021).

The *Pannonian* (~ 11.6–7.2 Ma, Fig. 2) interval is characterised by the expansion and the final retreat of the brackish Lake Pannon from the Vienna Basin (Magyar et al. 1999). The Pannonian succession includes siliciclastic lacustrine and terrigenous deposits, up to 1200 m thick (Harzhauser et al. 2004). The lower to middle Pannonian succession is characterized by lacustrine marls (Bzenec Fm.) and delta lobes of the paleo-Danube (Hollabrunn-Mistelbach Fm.) (Harzhauser et al. 2022). The upper Pannonian interval is dominated by a terrigenous wetland setting and is characterised by lignite seams in its basal part Čáry Fm.) and by sandy-marly facies in its upper part (Gbely Fm.) (Harzhauser and Tempfer 2004; Harzhauser et al. 2004). Uppermost Pannonian deposits are missing in large areas and may indicate post-Pannonian erosion of about 400 m in large parts of the Vienna Basin (Harzhauser et al. 2022).

### Petroleum system

The petroleum system in the Vienna Basin is mainly sourced by the upper Jurassic Mikulov Formation in the autochthonous unit (Ladwein 1988; Geršlová et al. 2015; Rupprecht et al. 2017). Reservoirs are present within all three tectonostratigraphic units, but most relevant for the present study are a large number of reservoir horizons found in transgressive and regressive sandstones in the Miocene basin fill. In the Matzen Field, hydrocarbons are produced from nine lower Miocene, 25 middle Miocene and four upper Miocene horizons (Arzmüller et al. 2006). Lower Miocene reservoir sands are usually elongated lens-shaped bodies of basinal or delta-plain origin isolated by shale. Middle Miocene reservoirs have been deposited in transgressive shoreline settings (Matzen Fm.), and in delta-front and delta-slope settings, while turbidite reservoir sandstones are rare. Upper Miocene (Pannonian) reservoir sands were deposited in delta-plain and delta-front environments. Sarmatian and Pannonian reservoirs typically host significant amounts of gas and minor oil. Seal rocks are represented by thick packages of fine-grained clastic rocks deposited during maximum flooding events, or mudstones covering single delta lobes.

Hydrocarbon traps in the Austrian part of the basin are related to major faults (e.g. Steinberg Fault), the central highs including the pre-Neogene floor (Aderklaa and Matzen), and the southern and SE fault systems (Arzmüller et al. 2006). Buried hills and thrust-internal traps exist in the Calcareous Alps.

Hydrocarbon generation occurred during overthrusting and Miocene basin subsidence. Because of its location directly above the mature source rock, the presence of large vertical faults, and the high number of vertically stacked reservoirs, the Vienna Basin petroleum system is classified as vertically drained. In the past, reservoir charge was thought to occur mainly along the major faults (Ladwein 1988; Arzmüller et al. 2006). However, the timing of hydrocarbon generation and migration suggests a second migration process possibly directly through the semi-permeable mudstone seal layers (Misch et al. 2021).

## Samples and methods

### Samples

The study is based on 68 core samples (Supplementary material Table 1) taken from 24 wells roughly aligned along a 30 km long NE-SW profile section in the Vienna Basin (Fig. 1b). The sampled cores originate from wells that were partly drilled in the 1970s and hence have been stored exposed to air for several decades. Bensing et al. (in press) conducted a comparative study of old vs. fresh, preserved core material from the Pannonian succession in the Vienna Basin to test the suitability of stored core material for pore structural investigations. Mudstone porosity determined with multiple porosimetry techniques was found to be comparable for stored and fresh core material from the same depth and stratigraphy, which confirms the applicability of stored core material for a porosity-compaction study. The investigated samples are Miocene calcareous mudstones (11 Pannonian, 13 Sarmatian, 44 Badenian) covering a depth interval from 720 to 3270 m (Fig. 1c). All samples were taken from several meter-thick mudstone intervals, to capture pure mudstone compaction processes rather than influences from mixed lithology. Therefore, other lithologies like Leitha limestones or evaporites from the middle Badenian were not considered. The well locations are Hohenau, Dobermannsdorf, Zistersdorf, Spannberg, Bockfliess and Aderklaa. Bulk mineralogy via X-ray diffraction (XRD), Rock–Eval parameters, and broad ion beam—scanning electron microscopy (BIB-SEM) were measured for all samples, while mercury injection capillary porosimetry (MICP) and He-pycnometry were applied to a sub-set of 41 samples.

### Methods

X-Ray diffraction, BIB-SEM, Eltra, and Rock–Eval measurements were performed in the labs of the chair of Petroleum Geology at Montanuniversität Leoben. Mercury injection capillary porosimetry (MICP) and He-pycnometry experiments were conducted at the petrophysical laboratory at Friedrich-Alexander University Erlangen-Nürnberg.

#### X-ray diffraction

Bulk mineralogical investigations via XRD were performed using a Panalytical X’Pert^3^ Powder diffractometer on texture-free powder samples. Samples were ground by hand to a grain size of approximately 10 μm. Measurements utilized CuKα-radiation (11.54 Å, 45 kV, 40 mA), and mineral quantification followed the method of Schultz (1964). Goniometer speed rate was set to 0.5°2*θ*/minute and a registration range from 2 to 66°2*θ* was used. The quantified mineral phases included quartz, plagioclase, potassium feldspar, calcite, dolomite, pyrite, siderite, and bulk clay minerals.

#### Helium pycnometry

Skeletal density (*ρ*_*s*_) of the selected samples was determined using Micromeritics AccuPyc II 1345 and helium gas. Sample weights and volumes ranged from 5.38–20.88 g to 1.97–7.63 cm^3^. With the bulk density (*ρ*_*b*_) from MICP measurements a porosity value *Φ*_He_ was calculated (Hedenblad 1997; Krus et al. 1997)

1 $${\Phi }_{\mathrm{He}} = \frac{{\rho }_{s}-{\rho }_{b}}{{\rho }_{s}}$$

#### Mercury injection capillary porosimetry

**Fig. 3:**
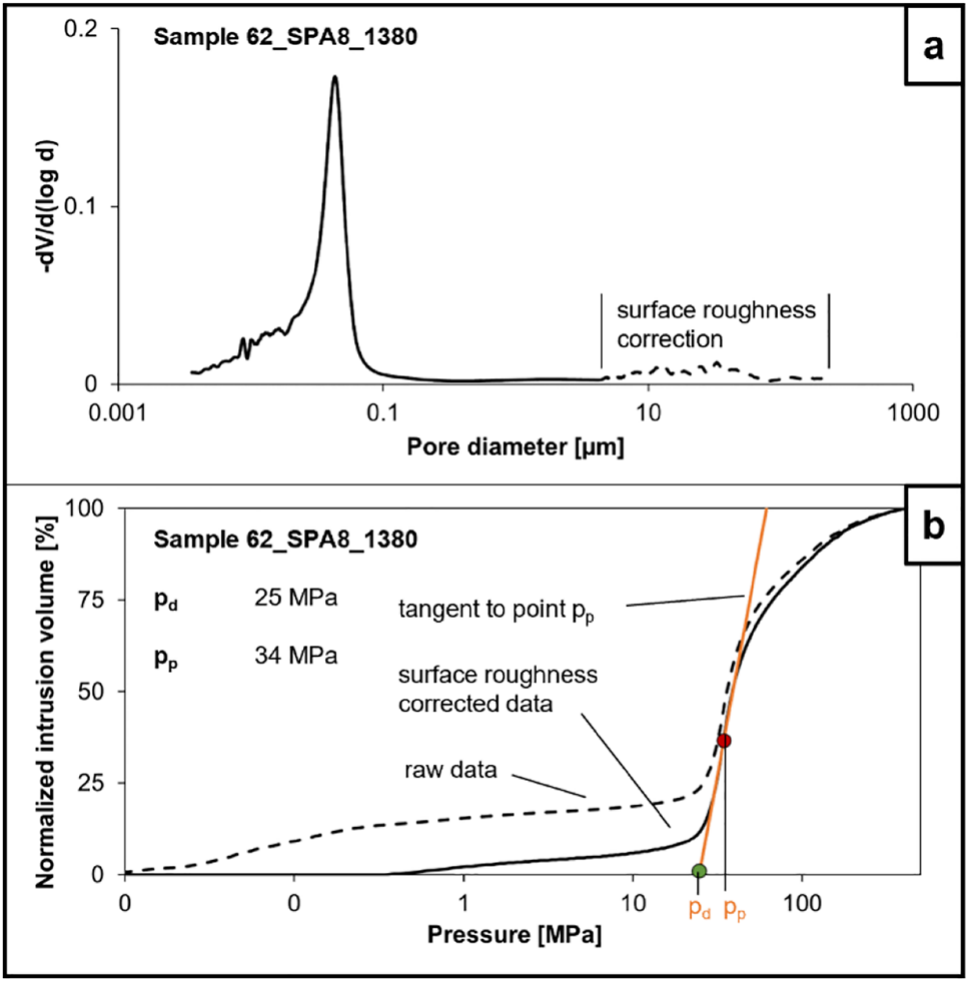
Example of typical Hg-injection curves (sample 62_SPA8_1380) that were used for surface roughness correction. The dotted line in (**a**) was removed from the total curve, by picking the entry pressure manually as it is interpreted as filling of irregularities of the sample surface (Amann-Hildenbrand et al. 2013). Note the irregular shape of the part that needs correction and the smooth outline of the following part. **b** A tangent was fitted to the inflection point (*p*_*p*_) of the injection curve to obtain the displacement pressure (*p*_*d*_)

For MICP measurements a Quantachrome Poremaster 60 apparatus was used. The machine generates pressures of up to ~ 400 MPa (60,000 psi). Sample weights and volumes ranged 1.69–2.66 g and 0.71–1.08 cm^3^, respectively. The sample material was oven-dried for 12 h at 105 °C prior to analysis. To obtain reliable pore throat radii and porosity values, capillary pressure curves were corrected for surface roughness by picking the entry pressure manually, assuming the irregularly shaped part of the pore throat distribution is not reflecting the true entry pressure (Busch and Amann-Hildenbrand 2013) (Fig. 3). The picked entry pressures ranged from 0.24 to 0.52 MPa. Afterwards, a tangent was fitted to the inflection point of each corrected curve to obtain the displacement pressure and the corresponding displacement pore throat radius.

#### Broad ion beam—scanning electron microscopy (BIB-SEM)

Samples for BIB-SEM investigation were cut with a Buehler IsoMet Low-Speed Precision Cutter to minimize mechanical disintegration. Water was used as a cooling agent/lubricant.

BIB-polishing was performed using a Hitachi ArBlade 5000 system (3 h at a milling energy of 8 kV). Samples were then coated with gold using a Cressington Sputter Coater 108auto (30 s sputtering time).

SEM image acquisition was done using a TESCAN CLARA field emission (FE) microscope equipped with the TESCAN Essence Image Snapper software (version 1.0.8.0). Images were taken at 10 kV electron energy. The mapping routine for each sample included (i) one large area map at a size of ~ 1000 × 1000 µm and 4000 × magnification (C1), as well as (ii) three detail maps at a size of ~ 200 × 200 µm and 20,000 × magnification (C2-4), corresponding to a pixel resolution of 13.7 nm. The image-based pore quantification was solely performed on the three detailed maps; pores smaller than 2 × 2 pixels were excluded from the pore statistics, resulting in a quantifiable minimum pore size of ~ 30 nm in equivalent pore diameter (Klaver et al. 2012; Houben et al. 2013; Mathia et al. 2019).

Pore segmentation was done with the pixel classification workflow of Ilastik version 1.3.3post3 (Shi et al. 2023). The segmented pore masks were analysed with Fiji for size and geometry distributions of SEM-visible porosity larger than the practical resolution of 30 nm. Pore masks have been slightly crack-corrected by hand.

#### Grain size estimation

An estimation of the equivalent diameter of the 50 largest grains was done by hand as it was not possible to segment the grains with an artificial intelligence-based algorithm because of overlapping grain boundaries. The measurements were done on the overview maps C1 to cover a large area. The largest detected grain (*ø*_max_) and an arithmetic mean of the 50 largest grains (*ø*_max50_) were used as semi-quantitative grain size parameters.

#### Normal compaction trends

Normal compaction trends neglecting abnormal pore pressure influence were used in numerous previous studies to establish porosity and paleo-burial models for mudstones in the zone of predominant mechanical compaction (Athy 1930; Baldwin et al. 1985; Drews et al. 2018; Ewy et al. 2020). Considering the often-existing direct relationship between porosity and permeability or displacement pressure (Yang and Aplin 2007, 2010), normal compaction trend curves may also serve as a seal quality prediction tool in areas lacking direct information from drill cores. However, the generalized trends need to be calibrated ideally for every basin. To test their general applicability as a model for displacement pressure and resulting maximum hydrocarbon column height, assuming exclusively capillary displacement, compaction vs. porosity/permeability trends derived from Yang and Aplin (1998, 2004, 2007, 2010) were compared to measured displacement pressures from MICP experiments and measured clay mineral contents from XRD. Note that the original model uses clay content as the proportion of clay-sized particles, whereas this study uses clay mineral contents from the XRD analysis to display the measured data. The necessary steps to calculate displacement pressure and HCH for mudstones of a given clay content and at a given burial depth after Yang and Aplin (1998, 2004, 2007, 2010) are described in the following. In the first step porosity is calculated from classical effective stress principles of soil mechanics (e.g., Terzaghi 1943; Skempton 1969; Burland 1990):

2 $$\phi { } = { }\left( {\frac{e}{1 + e}} \right)$$

3 $$e = e_{100} - \beta {\text{ln}}\left( {\frac{{\sigma^{\prime}_{v} }}{100}} \right)$$

where $\phi$ is porosity, $e$ is the void ratio, ${e}_{100}$ is the void ratio at 0.1 MPa effective stress and $\beta$ is the slope of the linear relation between the void ratio and the natural logarithm of vertical effective stress. The input parameters ${e}_{100}$ and $\beta$ are derived from an empirical relationship between void ratio and vertical effective stress, based on the assumed clay content (Yang and Aplin 2004):

4 $$e_{100} = 0.3024 + 1.6867\;clay + 1.9505\;clay^{2}$$

5 $$\beta = 0.0407 + 0.2479\;clay + 0.3684\;clay^{2}$$

following the definition of clay as being the mass fraction of particles < 2 µm in diameter (Yang and Aplin 2004).

Utilizing an empirical relationship of porosity vs. clay content, the vertical permeability can be derived:

6 $$\begin{aligned} \ln \left( {K_{v} } \right)& = - 69.59 - 26.79*clay + 44.07*clay^{{0.5~}} \\ & \quad\, + \left( { - 53.61 - 80.03*clay + 132.78*clay^{{0.5~}} } \right)*e \\ & \quad\, + \left( {86.61 + 81.91*clay - 163.61*clay^{{0.5~}} } \right)*e^{{0.5~}} \\ \end{aligned}$$

where $K_{v}$ is the bedding perpendicular permeability.

**Fig. 4:**
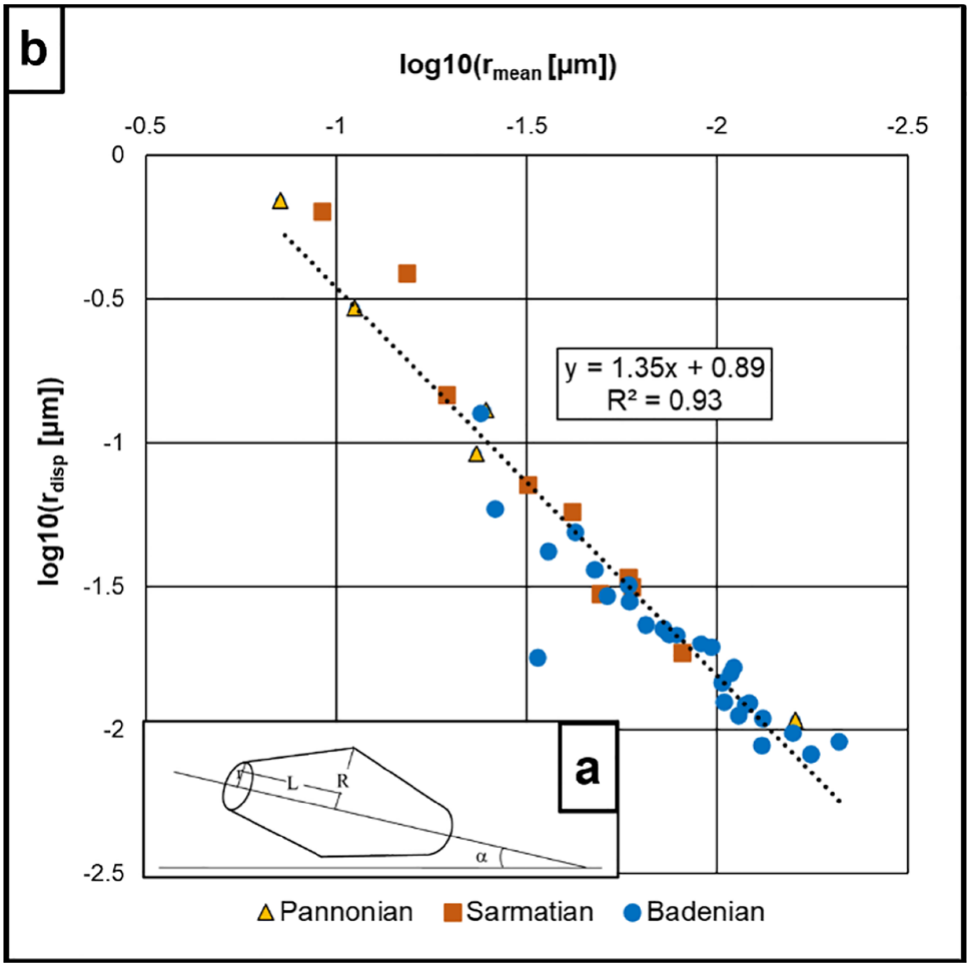
**a** Assumed pore shape from Yang and Aplin (1998). *R* is the widest radius of the pore body, *r* is the pore throat radius, *L* is the length from the widest radius to the pore throat *r* and $\boldsymbol{\alpha }$ is the alignment angle. (modified from Yang and Aplin 2007). **b** Mean pore throat radius (*r*_mean_) vs displacement radius (*r*_disp_). The correlation of mean pore throat radii and displacement radii (*R*^2^ = 0.93) is used to transform mean radii from the Young and Aplin model into displacement radii

Applying the pore throat model of Yang and Aplin (1998) (Fig. 4a) and using the relationship of the calculated porosity and permeability values established by Yang and Aplin (2007), it is possible to calculate the mean pore throat radius:

7 $$r_{m} = \left( {\frac{{K_{v} }}{{10^{ - 19,21} *J_{v}^{1.118} }}} \right)^{{\frac{1}{1.074}}}$$

8 $$J_{v} = \frac{9}{8}*\phi *\left( {\sin \left( \alpha \right)} \right)^{2} *\frac{{J_{1}^{3} }}{{\left( {1 + J_{1} + J_{1}^{2} } \right)^{2} }}$$

9 $$J_{1} = 2.371 - 1.626*clay^{2 } + 153.8*\phi^{4}\, \quad\, J_{1} = \frac{R}{r}$$

10 $$\alpha = 45^\circ - 10.24^\circ *\left( {e_{100} - e} \right)\, \quad\, \sigma^{\prime} > 100kPa$$

where $J_{1}$ is the ratio of the largest radius of a pore to its throat radius, supposed to be equal for all pores of a sample. $\alpha$ is the average pore alignment angle relative to the bedding direction.

#### Maximum hydrocarbon column height estimation

The Yang and Aplin model establishes a compaction trend for mean pore throat radii. However, to calculate HCH the mean pore throat radii (*r*_mean_) need to be converted to displacement radii (*r*_disp_). This was done by applying the following logarithmic correlation derived from the measured data of this study:

11 $$\log 10(r_{{{\text{disp}}}} ) = 1.35 \log 10\left( {r_{{{\text{mean}}}} } \right) + 0.89\quad\, R^{2} = 0.93$$

which is derived from the measured capillary pressure curves (Fig. 4b). This displacement radius is then used to calculate a theoretical maximum hydrocarbon column height (HCH) by equating the buoyancy pressure with the capillary entry pressure of the pore:

12 $$\mathrm{HCH}=\frac{2*{\sigma }_{\mathrm{inter}}*\mathrm{cos}(\theta )}{{r}_{\mathrm{disp}}\left({\rho }_{\mathrm{water}}-{\rho }_{\mathrm{oil}}\right)*g}$$

where ${\sigma }_{\mathrm{inter}}$ is the interfacial tension of the fluid (0.035 N/m), $\theta$ is the wetting angle of the sample (0°), ${\rho }_{\mathrm{oil}}$ is the hydrocarbon density (0.807 g/cm^3^), ${\rho }_{\mathrm{water}}$ is the water density (1.06 g/cm^3^) and g is the gravity constant (9.81 m/s^2^) (Schowalter 1979; Yang and Aplin 2010). It must be noted that the theoretical hydrocarbon column heights reflect pure static capillary sealing behaviour, while dynamic effects as well as seal heterogeneities or discontinuities are ignored. Hence calculated values > 1500 m HCH occur frequently in the presented data set, clearly pointing to an overestimated practical seal capacity.

For the samples only measured with BIB-SEM, HCH was calculated using the regression line derived from the correlation of *Φ*_SEM_ and HCH of this study:

13 $$\mathrm{HCH}=7.7062*{\Phi \mathrm{SEM}}^{-1.427} \quad {R}^{2}=0.68$$

#### Eltra/rock–eval

Carbon and sulphur measurements were performed twice on powdered rock samples, using an Eltra Helios analyser. Total sulphur (S), total carbon (C) and total organic carbon (TOC, after treatment with phosphoric acid to remove inorganic carbon e.g., carbonate) were determined. Samples were pyrolyzed with a “Rock–Eval 6” instrument (Vinci Technologies). With the measurements of the S1 and S2 peaks [mg_HC_/g_rock_] the Production-Index [PI = S1/(S1 + S2)] was calculated (Espitalié et al. 1977). S1 is sensitive to free hydrocarbons present in the rock and S2 represents hydrocarbons that were generated during pyrolysis. The Production-Index represents the amount of hydrocarbons generated from the total organic matter present in the rock. As more hydrocarbons are generated with increasing temperature and therefore depth, an increasing depth trend would be expected. Considering the negligible primary source potential of the samples, S1 and PI were mainly used to identify the amount of hydrocarbon staining in the samples. *T*_max_ and S1 values of samples with an S2 value < 0.25 mg_HC_/g_rock_ (4 samples) were excluded from the interpretations due to obvious measurement bias.

## Results

Bulk mineralogical, He-pycnometry, MICP, BIB-SEM and bulk geochemical data are displayed in Tables 2, 3, 4 and 5 in the supplementary.

### Bulk mineralogy

**Fig. 5:**
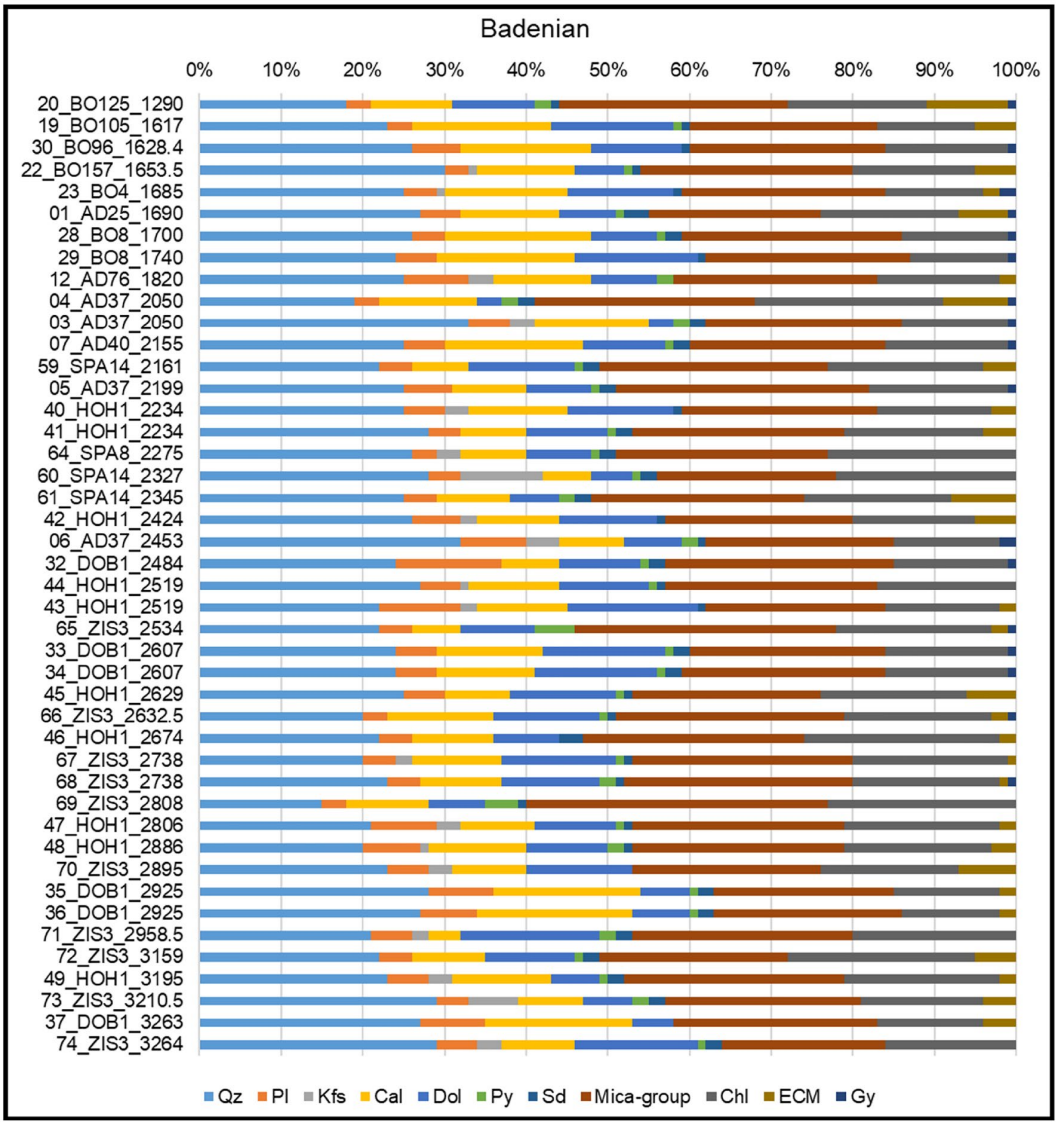
Bulk mineralogic analyses of Badenian samples. Samples are aligned according to their depth (top = shallowest). Results are given in wt%. Main phases: Mica-group-minerals, quartz chlorite, dolomite, and calcite. In minor to trace amounts plagioclase, expendable clay minerals, gypsum, potassium feldspar, pyrite and siderite were detected. Badenian samples tend to include more calcite than dolomite and contain less plagioclase compared to the other formations. (Qz quartz, Pl plagioclase, Kfs K-feldspar, Cal calcite, Dol dolomite, Py pyrite, Sd siderite, Chl chlorite, ECM expandable clay minerals, Gy gypsum)

**Fig. 6:**
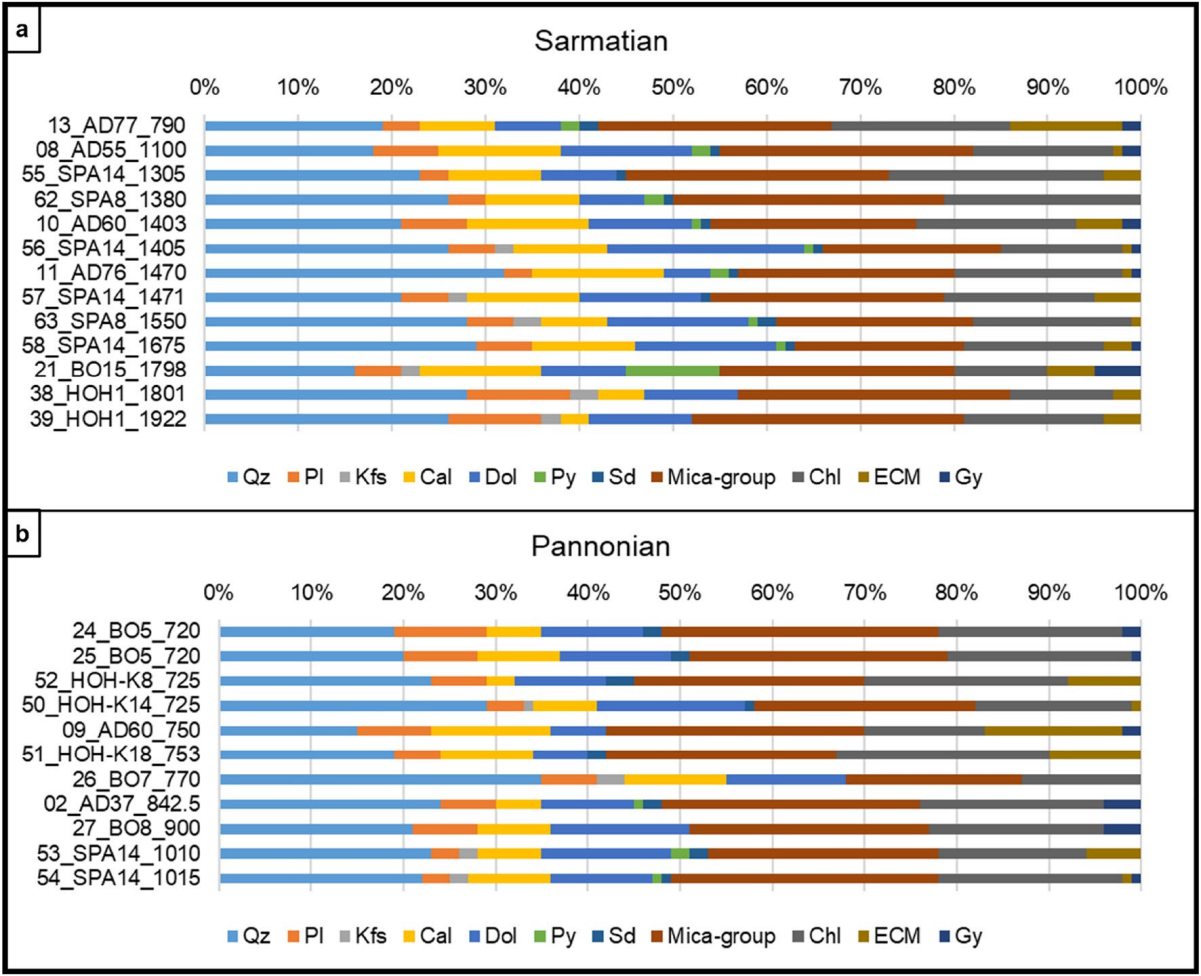
Bulk mineralogical analyses of Sarmatian (**a**) and Pannonian (**b**) samples. Samples are arranged according to their depth (top = shallowest). Results are given in wt%. Main phases: mica-group-minerals, quartz chlorite, dolomite, and calcite. With minor to trace amounts plagioclase, expandable clay minerals, gypsum, potassium feldspar, pyrite and siderite were detected. Pannonian samples are generally slightly richer in clay minerals, but no correlation between sample depths and clay mineral amounts has been observed. (Qz quartz, Pl plagioclase, Kfs K-feldspar, Cal calcite, Dol dolomite, Py pyrite, Sd siderite, Chl chlorite, ECM expandable clay minerals, Gy gypsum)

The following major mineral phases are quantified by XRD analyses (Figs. 5 and 6): mica group minerals (18–37 wt%), quartz (15–35 wt%), chlorite (10–24 wt%), dolomite (6–21 wt%), and calcite (3–18 wt%). Furthermore, minor to trace amounts of plagioclase (3–11 wt%), expandable clay minerals (0–10 wt%), gypsum (0–5 wt%), potassium feldspar (0–6 wt%), pyrite (0–10 wt%), and siderite (0–3 wt%) are detected in parts of the samples.

Only minor stratigraphic differences in bulk mineralogy are observed; Badenian samples tend to include slightly more calcite and less plagioclase compared to the Sarmatian and Pannonian samples. Furthermore, Pannonian samples are slightly richer in bulk clay minerals, but no depth trend of clay mineral is visible (Figs. 5 and 6).

### Helium and mercury intrusion porosimetry

**Fig. 7:**
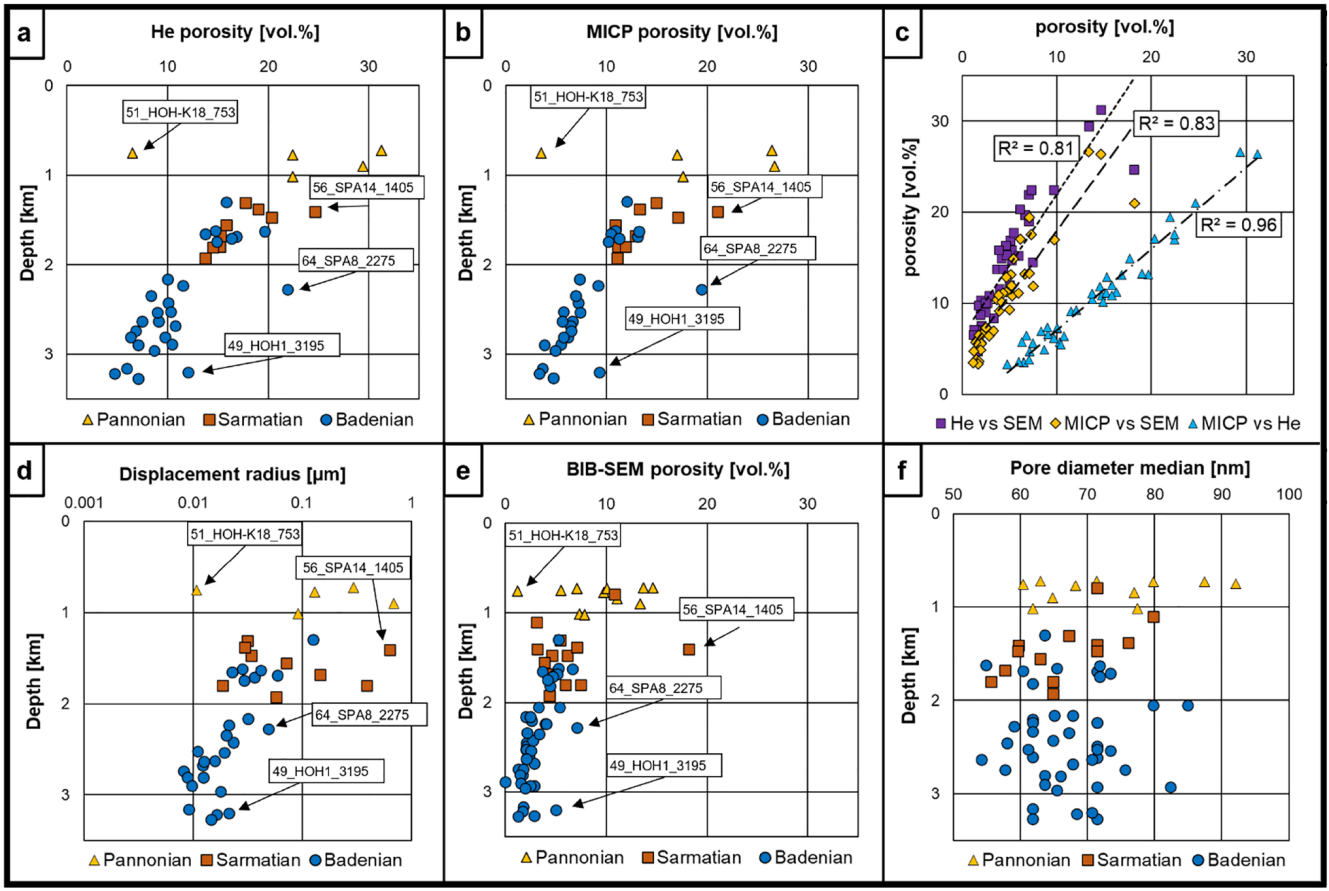
**a** Total porosity values determined by He-pycnometry plotted vs. depth (note that samples are color-coded according to stratigraphy). **b** Total porosity values determined by MICP plotted vs. depth. **c** Correlations of the different porosity methods. The three methods are well comparable for this sample set, although *Φ*_He_ values show a systematical shift towards higher porosities and ΦS_EM_ values are systematically lower. **d** Estimated displacement radii from capillary pressure curves plotted vs. depth. Note that the displacement radius axis is in logarithmic scale. In general, the displacement radii are decreasing with depth. One shallow Pannonian sample does not follow this general trend and reaches a remarkably low displacement radius. **e** Total porosity values determined by BIB-SEM plotted vs. depth. **f** Semilog plot of BIB-SEM mean pore diameters vs displacement radii, showing a weak correlation

Corrected bulk density values of dry samples range at 1.87–2.61 g/cm^3^, while the corrected Φ_MICP_ values show a wide range from 3.3 to 26.7 vol.% reflecting the broad sampled depth interval. MICP porosity values are systematically lower compared to porosity values derived from He pycnometry, which range from 4.7 to 31.2 vol.% (Fig. 7a, b; Supplementary material Table 3). MICP is restricted to pore throats >  ~ 3 nm compared to 1.2 nm in the case of He-pycnometry (Freitag et al. 2022) which results in the observed systematic shift in detectable porosity. Both *Φ*_MICP_ and *Φ*_He_ show decreasing trends with depth, with the shallower Pannonian samples exhibiting the highest and the deepest Badenian samples exhibiting the lowest porosity values for each method. Values for *Φ*_He_ and *Φ*_MICP_ show a good correlation (*R*^2^ ~ 0.96; Fig. 7c), which indicates reliable capillary pressure data despite the low-permeability nature of the investigated samples, that need to be checked more carefully compared to reservoir-type rocks such as sandstones (Busch and Amann-Hildenbrand 2013; Houben et al. 2014; Klaver et al. 2017). Displacement radii range from 0.0043 to 0.2135 µm and show a decreasing depth trend comparable to that of *Φ*_MICP_ (Fig. 7d). The Pannonian samples show the highest variability of displacement radii (0.0048–0.2135 µm), whereas Sarmatian and Badenian samples generally show radii < 0.05 µm and 0.02 µm, respectively. Mean pore throat radii (*r*_mean_) range from 0.0048 to 0.138 µm. Displacement pressures (*p*_disp._) are between 3 and 172 MPa, whereas the saturation at breakthrough (*V*_disp._) ranges from 36–57%. The shallow Pannonian samples show a wider porosity scattering, while a well-defined depth trend can be seen for Sarmatian and Badenian samples in both porosity data sets (Fig. 7a, b). Only a few outliers are observed, which are partly a result of varying sedimentological characteristics or cementation patterns. Sample 51_HOH-K18_753 is strongly calcite-cemented and, therefore, shows a very low porosity for its depth position. Sample 56_SPA14_1405 was erroneously collected from a fine sand interval intercalating with the target mudstone. It shows a distinctly larger displacement pore throat radius (Supplementary material Table 3) confirming its coarse-grained nature. Samples 64_SPA8_2275 and 49_HOH1_3195 from depths > 2000 m contain comparably higher silt contents, possibly resulting in less effective mechanical compaction compared to clay-rich samples.

### Scanning electron microscopy

The image-based *Φ*_SEM_ values vary between 1.18 and 18.21%, which is considerably lower compared to *Φ*_He_ and *Φ*_MICP_, due to the practical resolution limit of 30 nm for the given mapping workflow (Supplementary material Table 4). However, *Φ*_SEM_ shows a strong correlation with both *Φ*_MICP_ and *Φ*_He_ with *R*^2^ values of 0.83 and 0.81 (Fig. 7c), respectively, as well as similar porosity-depth trends (Fig. 7a, b, e). Excluding the fine sandstone sample 56_SPA14_1405 increases *R*^2^ to a value of 0.90 for the correlation of *Φ*_SEM_ with both *Φ*_He_ and *Φ*_MICP_.

**Fig. 8:**
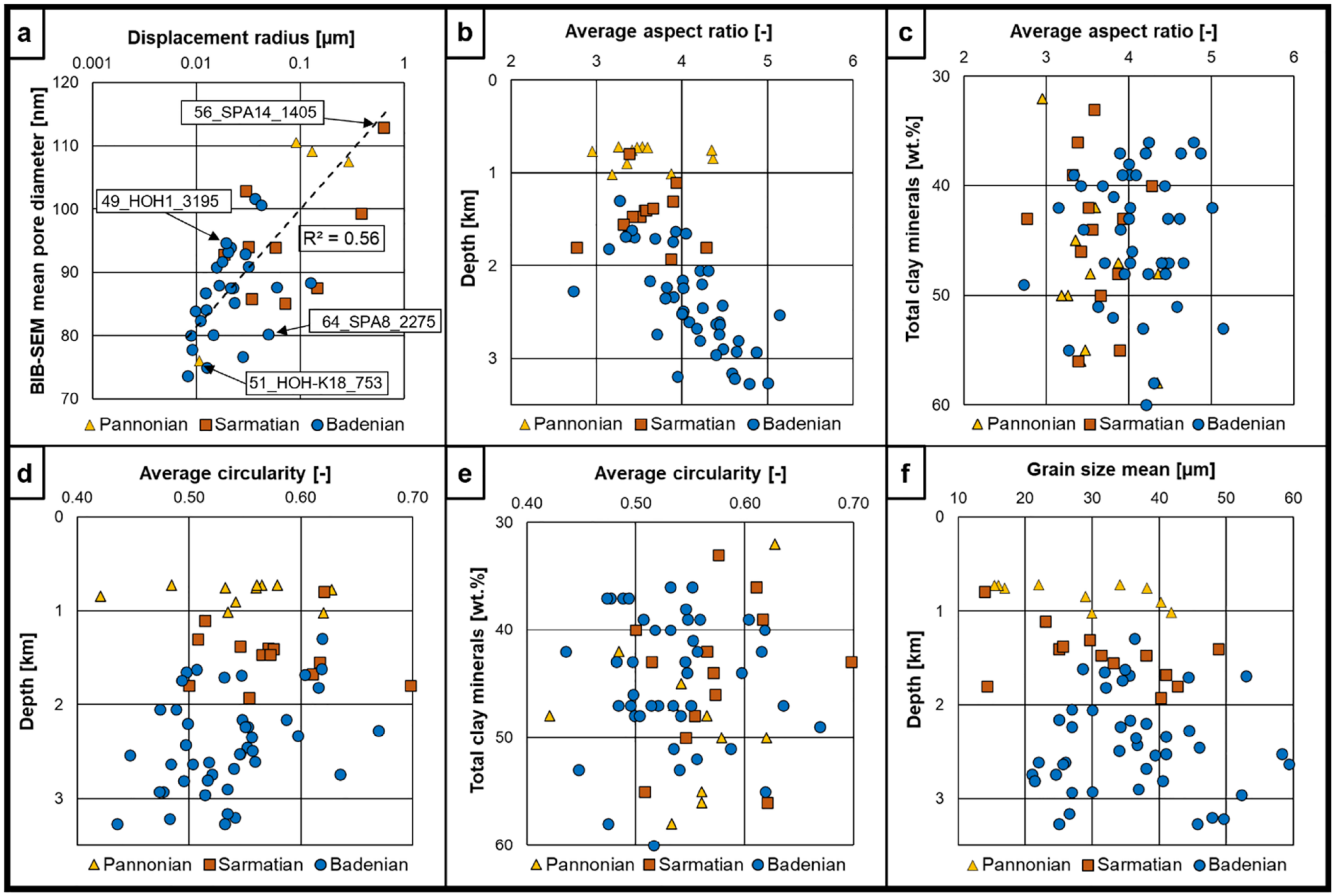
**a** Semilog plot of BIB-SEM mean pore diameters vs displacement radii, showing a weak correlation. **b** Average aspect ratios (AR) vs. depth. A slight trend to higher AR’s with increasing depth is visible. **c** Average AR’s vs. TCM. Slight correlation of higher AR’s with increasing clay mineral contents, but clay mineral contents do not correlate with depth. **d** Average pore circularities vs. depth. Pore shapes get more complex with increasing depth. **e** Average circularity vs TCM. **f** Mean grain sizes vs. depth. Both pore diameters and grain sizes do not correlate with depth. This indicates that the proposed depth trends are not influenced by geological variations

BIB-SEM offers the opportunity to use geometry parameters directly derived from segmented pore cross-sections for pore shape description. Median pore diameters segmented from BIB-SEM maps range from 54 to 77 nm and do not correlate with depth (Fig. 7f). The displacement radii determined from MICP show a moderate trend with the BIB-SEM mean cross-sectional pore diameter (*R*^2^ ~ 0.60; Fig. 8a). The average aspect ratios (2.73–5.14) of segmented pores show an increasing depth trend (Fig. 8b), while there is no correlation with the bulk amount of clay minerals (Fig. 8c). Furthermore, the average pore circularities (0.45–0.70) show a very weak decreasing depth trend and no correlation with total clay minerals (Fig. 8d, e).

**Fig. 9:**
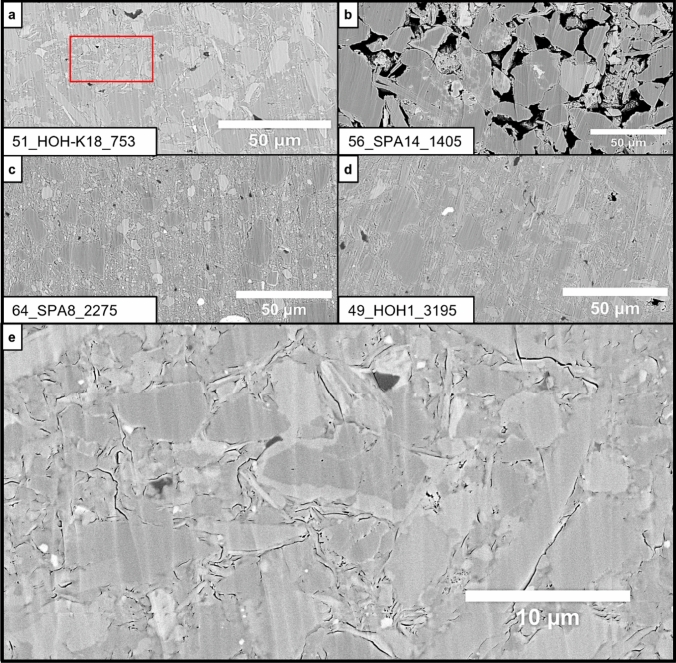
BIB-SEM images of samples that do not fit into the general porosity depth trend. **a** Sample 51_HOH-K18_753 shows very low porosity values for its depth position (755 m, 6.51 vol. % Φ_He_). The image reveals a strong carbonate cementation of the pore structure, leading to a lower porosity value. A detail image of the cementation is displayed in (**e**) (red rectangle marks the position of the presented element).** b** Sample_56_SPA14_1405 possesses an abnormally high porosity for its depth position (1406.5 m, 24.64 vol. % *Φ*_He_). The microstructure image confirms that this is no analytical bias but rather since a fine sandstone was erroneously sampled. **c** and **d** Samples with a coarser grain size that results in a higher as expected porosity. **e** Detail of red rectangle in (**a**) revealing strong carbonate cementation of the pore structure

The semi-quantitative grain size parameters *ø*_max_ and *ø*_max50_ derived from the overview image maps range from 26 to 330 µm and from 14 to 59 µm, respectively, and do not show a correlation with depth or a systematic shift according to the stratigraphic intervals (Fig. 8f). According to the BIB-SEM overview maps, most of the samples are matrix supported and evidence for significant carbonate diagenesis and associated calcite cementation was observed exclusively in one shallow sample (51_HOH-K18_753; Fig. 9). Minor calcite cementation was otherwise only observed in the deepest samples. However, these samples still follow the overall porosity-depth trends (Fig. 7a, b, e).

### Maximum hydrocarbon column heights based on capillary pressure curves

**Fig. 10:**
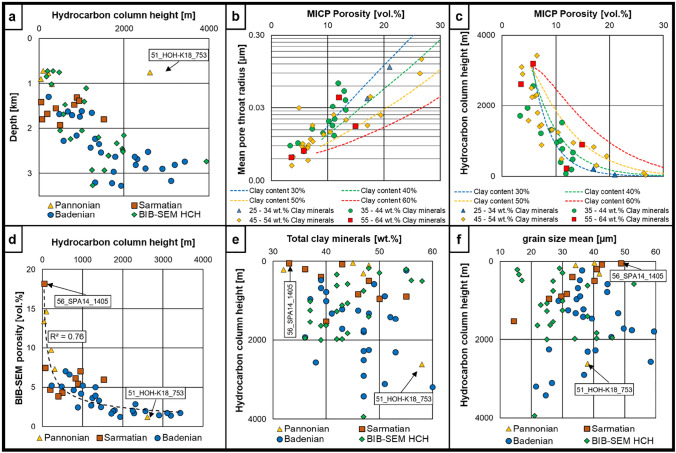
**a** Estimated maximum HCH plotted vs. depth. The observed HCH depth trend argues for a normal compaction for most of the investigated samples (note that samples are color-coded according to stratigraphy). **b** Mean pore throat radii plotted vs. *Φ*_MICP_ **c** HCH plotted vs. *Φ*_MICP_. Dashed lines in (**b**) and (**c**) are calculated based on models of Yang and Aplin 1998, 2004, 2007, 2010. **d** *Φ*_SEM_ vs HCH. Note that a potential correlation has been chosen because the calculation of the HCH only considers the radii and the porosity the square of the radii. **e** Total clay mineral contents plotted vs. HCH’s. **f** Image-based grain size means plotted vs. HCH’s. HCH are neither correlating with total clay mineral contents nor with grain size means derived from SEM-images. BIB-SEM HCH = Hydrocarbon column heights calculated with the correlation of BIB-SEM mean pore diameter and displacement radius derived from MICP

The calculated maximum HCH values vary between 132 and 6612 m, following a depth trend with the lowest values seen in the shallowest Pannonian and the highest values in the deepest Badenian intervals (Fig. 10a). The mean pore throat radii and calculated HCH values derived from capillary pressure curves generally show good fits with the calculated trends based on mathematical compaction models (Fig. 10b, c). As can be seen in Fig. 10b, c the bulk clay mineral contents from XRD do not agree with the modelled clay content lines. Note that clay minerals can be larger than clay grade and a general correlation of those two parameters is not given. Nevertheless, the clay fraction trends seem to be slightly better represented by bulk clay mineral contents for shallower samples with higher *Φ*_MICP_ (> 10–15 vol.%) and less intense compaction, whereas the estimated seal capacities of low-porosity samples show no sensitivity to bulk mineralogy. HCH values plotted against *Φ*_SEM_ correlate with an *R*^2^ value of 0.80 (Fig. 10d).

Sample 51_HOH-K18_753 represents one exception with an extremely high HCH value despite its shallow depth; this is likely caused by an extremely high bulk clay mineral content and extensive calcite cementation of the clay matrix (Fig. 9a, e). This sample has a *Φ*_MICP_ of 1.18 vol.% and an average displacement radius of 0.0048 µm, ranging among the lowest values within the sample set.

### Bulk geochemical parameters

**Fig. 11:**
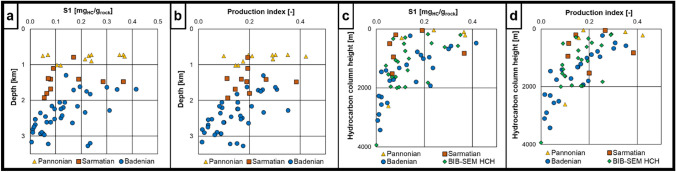
**a** Rock–Eval S1 plotted vs. depth. Although the signal intensity is low, a reverse depth trend is visible. **b** Rock–Eval PI plotted vs. depth, showing a reverse trend. Results from sample 22_BO157_1653.5 are not shown in the plot because of obvious measurement issues. S1 values are only shown if S2 > 0.25. (note that samples are color-coded according to stratigraphy) **c** Rock–Eval S1 plotted vs. HCH displaying a weak correlation of both parameters **d** Rock–Eval production index (PI) plotted vs. HCH, indicating a weak correlation of both parameters. BIB-SEM HCH = Hydrocarbon column heights calculated with the correlation of BIB-SEM mean pore diameter and displacement radius from MICP

The measured TOC contents are almost exclusively < 1 wt% except for the Pannonian sample 27_BO8_900 which contains 2.5 wt% TOC. S1 and S2 peaks show low absolute values after correction for background signal (S1: 0.03–0.46 mgHC/grock; S2: 0.2–1.3 mgHC/grock), indicating negligible primary source potential and potentially additional loss of volatiles during long-term storage. However, the signals are still strong enough to compare relative changes in S1 and PI (Supplementary material Table 5). S1 values plotted against depth indicate a slight reverse depth trend which is particularly visible in the Badenian sub-set of samples (Fig. 11a). PI values range from 0.07 to 0.45 and show a weak decreasing depth trend as well (Fig. 11b). Clearer trends of both S1 and PI were found compared with the calculated HCH values. Samples with lower HCH tend to have higher S1 and PI values and vice versa (Fig. 11c, d).

## Discussion

### Porosity depth trends

The core material used in this study was intentionally selected to cover large parts of the central Vienna Basin as well as a broad depth range within the middle Miocene succession including Badenian to Pannonian strata. The sample set hence covers varying depositional environments, which may have resulted in a certain spread of porosity data due to primary sedimentary signals (e.g., Misch et al. 2021). Most of the cores were drilled decades ago and stored under questionable preservation conditions, which is considered particularly problematic for mudstones (Ewy 2015). However, a recent comparative study on old vs. fresh Pannonian mudstone core material from the Vienna Basin showed that core material stored under air exposure may still be used for porosity studies provided its macroscopic integrity (Bensing et al. in press). These results as well as the good correlation between different porosimetry techniques observed in this study suggest a generally reliable data quality.

In the Vienna Basin, no pronounced overpressure zones are known and it is believed that the pore pressure is relatively close to hydrostatic conditions. Decreasing porosity-depth trends are clearly visible throughout all stratigraphic intervals and particularly obvious in the deepest Badenian but also the majority of Sarmatian samples (Fig. 7a, b, e). The continuous decreasing depth trends validate the assumptions of pore pressures close to hydrostatic conditions, as no systematic deviation of the trend was observed. Considering the regional sample coverage, this suggests a basin-wide similar compaction trend and porosity evolution (Fig. 7a, b, e), which was also noticed for the East Slovak Basin by Subová et al. (2022). Few outliers are rather a result of lithological variations than analytical bias. E.g., sample 51_HOH-K18_753 (755 m, 6.51 vol.% *Φ*_He_) does not follow the general compaction trend because of its strongly calcite-cemented clay matrix (Fig. 9a, e). Sample 56_SPA14_1405 is a fine sandstone that was erroneously sampled from a supposed mudstone core interval, and samples 64_SPA8_2275 and 49_HOH1_3195 from > 2000 m burial depth are silt-dominated, which likely led to less intense mechanical compaction of the clay matrix (Fig. 9b, c, d).

The shallow Pannonian samples display a wider scattering of porosity data, which may be further exaggerated by the limited number of samples from this stratigraphic unit. Furthermore, the shallowest samples with the highest initial porosities may have been most prone to drying-induced microstructural damage, although no strong drying effect on Pannonian core material was reported by (Bensing et al. in press). Overall, mineralogical variations seem to have a comparably minor influence on the porosity and pore throat distribution changes with depth, as inferred from the modelled clay content lines vs. the actually measured clay mineral contents shown in Fig. 10b, c. No trends of image-based grain size and pore shape data with bulk mineralogy were observed (Fig. 8), indicating that the porosities of the investigated sample set are primarily controlled by burial depth. Although all three porosimetry techniques show comparable depth trends, the determined absolute porosity values differ strongly due to the inherent detection range of each method (Supplementary material Tables 3 and 4). However, despite these shifts in measured absolute porosity values, the high correlation coefficients of *Φ*_SEM_ with *Φ*_He_ (0.81) and *Φ*_MICP_ (0.83), as well as the nearly perfect correlation of *Φ*_He_ with *Φ*_MICP_ (0.96) allow for the substitution of either one of the methods by another one, based on the established linear regressions (Fig. 7c). A slightly lower detectable pore size cut-off for He-pycnometry in comparison to MICP causes *Φ*_He_ to detect ~ 26% more pores compared to *Φ*_MICP_. The image-based *Φ*_SEM_ is restricted to a practical resolution of 30 nm, which causes it to capture only ~ 32% of the pores detected by He-pycnometry. This implies that most of the pore volume is contributed by pores < 30 nm in equivalent diameter, which agrees with previous studies on mudstone porosities in the Vienna Basin (e.g., Misch et al. 2021; Bensing et al. in press).

### Top seal quality estimation

Seal rocks are an essential element of active petroleum systems (Magoon and Dow 2009). However, while existing hydrocarbon accumulations previously often served as indirect proof for a working top seal in mature hydrocarbon provinces such as the Vienna Basin, fundamental research on low-permeable barrier layers became even more important in recent years in the context of nuclear waste disposal and underground gas storage in non-hydrocarbon reservoirs (Norris 2014; Espinoza and Santamarina 2017; Dewhurst et al. 2019; Ringrose and Meckel 2019; Tarkowski 2019; Heinemann et al. 2021; Bensing et al. 2022; Kivi et al. 2022; Nhabanga and Ringrose 2022; Vafaie et al. 2023). In addition, changes in top seal quality may affect hydrocarbon migration pathways particularly in vertically drained basins (Misch et al. 2021, 2022). Hence, an improved understanding of the regional top seal quality distribution will be crucial for future hydrocarbon exploration but also alternative underground storage activities in the Vienna Basin. In this study we calculated theoretical maximum HCH based on capillary displacement pressure values which were derived from capillary pressure curves (Fig. 10). These column heights are valid for oil of a given density and viscosity, assuming a water-wet seal. However, the calculated HCH values could easily be adapted to other pore fluids and physical parameters of the seal (Naylor et al. 2011). The experimental HCH vs. depth trend was compared with calculated column heights based on mathematical compaction models of Yang and Aplin (1998, 2004, 2007, 2010) (Fig. 10c). This comparison shows that the experimental vs. mathematical column height vs. depth trends are in good agreement. Yang and Aplin (2010) postulated a major influence of clay content, which could not be tested in this study due to the lack of “true” clay content data for the Vienna Basin mudstones. Nevertheless, no sensitivity of maximum hydrocarbon column heights to total clay mineral contents or SEM-based semi-quantitative grain size parameters was observed (Fig. 10e, f). For this set of relatively similar matrix-supported mudstones with clay contents > 30–35%, the main influencing factor on pore parameters (e.g., pore throat distributions) is the compaction-induced loss of porosity with depth (effective stress), while the clay mineral content is likely only a secondary influence (Figs. 7a, 10a).

The plots of *Φ*_MICP_ against mean pore throat radii and HCH values shown in Fig. 10b, c suggest a relatively homogeneous trend of increasing capillary sealing capacity with depth. However, many samples show theoretical column heights > 1500 m, which is clearly overestimated as maximum naturally observed column heights e.g. for the Norwegian continental shelf are between 300 and 700 m (Edmundson et al. 2021) and seals with a maximum column height > 1500 m are already classified as excellent (Sneider et al. 1997). That shows that additional field calibration would be required. However, this is not possible for the present data set since no real column height data are available for the Vienna Basin. Furthermore, due to its complex stacked reservoir architecture, other charging factors must be taken into consideration and column heights will not be purely top seal-controlled in the majority of cases were sampled intervals are located directly above hydrocarbon accumulations.

Displacement pore throat radii also follow a depth trend (Fig. 7d) and weakly correlate with the corresponding BIB-SEM mean pore diameters (*R*^2^ ~ 0.60), while *Φ*_SEM_ shows a strong correlation with hydrocarbon column heights derived from MICP (*R*^2^ ~ 0.80) (Figs. 8d and 10d). Therefore, BIB-SEM parameters may potentially be used as an approximation for capillary pressure curves in cases where core petrophysical data are not available (e.g., wells with available cuttings; see also Misch et al. 2021).

### Indications for vertical hydrocarbon migration

Rock–Eval parameters S1 and PI were used as indicators for free hydrocarbons in the sampled mudstones, following an approach introduced by Misch et al. (2022) for a known vertical top seal succession. Considering that all investigated samples are organic lean and that the shallower samples are thermally immature, these free hydrocarbons are interpreted as residues of oil staining which, according to the pyrograms, originates from a natural source (in contrast to e.g., oil-based mud during coring). As shown in Fig. 11c d, S1 and PI correlate well with the estimated HCH, particularly for the Badenian sub-set of samples. Furthermore, the PI and S1 values show a weak decreasing trend with depth (Fig. 11a, b), which contradicts a primary organic matter maturation trend where PI would increase with depth. This may indicate that hydrocarbons were vertically migrating through these semi-permeable layers, confirming the results of Misch et al. (2021). Although the measured data cannot quantify the rate of hydrocarbon displacement into the mudstone layers, they still point to a higher-than-expected vertical fluid mobility within the low-permeable seal lithologies. Given the considerable theoretical column heights derived from both MICP and mathematical models, it is surprising that apparently most of the shallower samples show oil staining from a supposedly natural (i.e., vertically migrating) source. Potential rock-fluid interactions (e.g., calcite dissolution; Flesch et al. 2018; Zou et al. 2018; Bensing et al. 2022; Labus and Tarkowski 2022) after displacement of e.g., CO_2_-enriched brines are often addressed as a potential long-term change factor for seal integrity. Considering the present discussion regarding top seal assessment for secondary gas storage, the implications of these findings must be critically reviewed in the abovementioned context in future studies.

### Implications for regional uplift and erosion

The extent of young uplift and erosion in the Vienna Basin area is a controversial issue because neither vitrinite reflectance data from the Miocene basin fill (unpublished data from Montanuniversitaet Leoben), nor thermochronological data (Heberer et al. 2022 and personal communication) yield clear results. Moisture contents of upper Pannonian lignite seams in the southern (Weber and Weiss 1983) and northern part of the Vienna Basin (Honěk et al. 2009) show that erosion was minor and probably did not exceed a few hundred meters (Heberer et al. 2022 and personal communication). Compaction trends of mudstones are applied in this section as an additional tool to quantify erosion. This seems reasonable as no pronounced overpressure zones exist in the Vienna Basin.

**Fig. 12:**
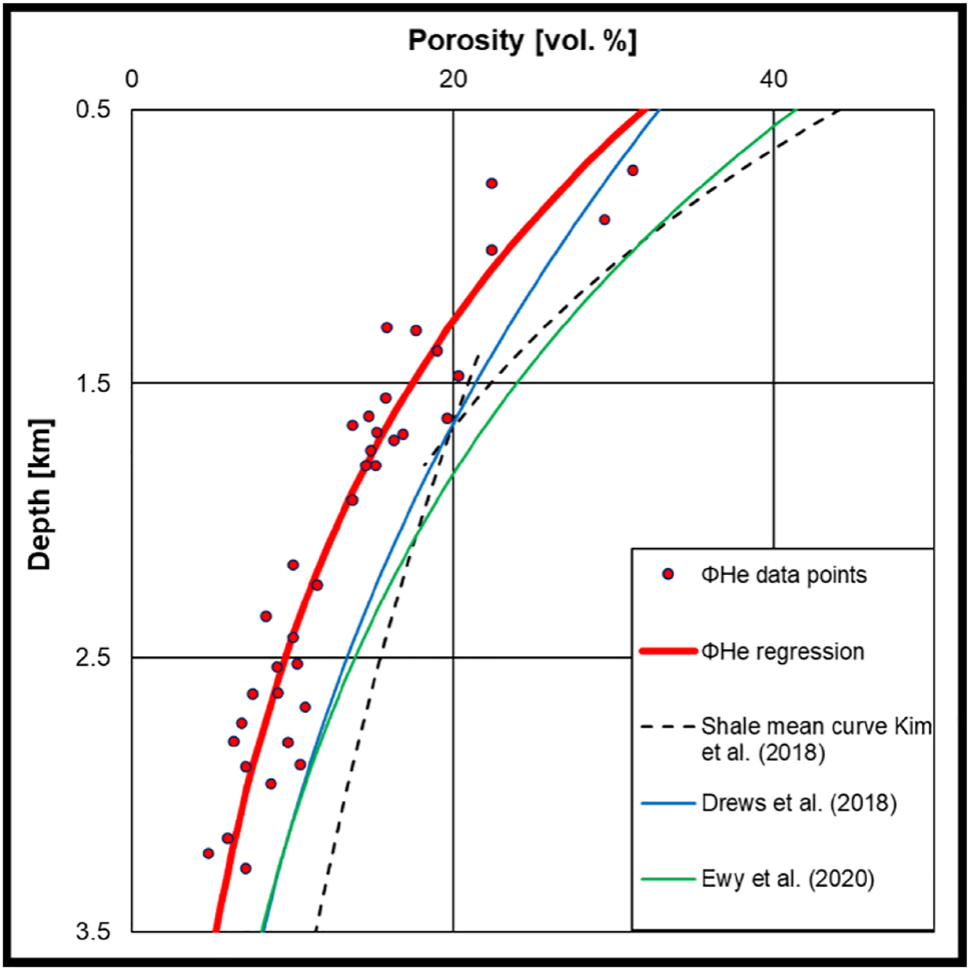
Comparison of the observed compaction trend with published compaction trends from different basins worldwide. Red dots show the measured *Φ*_He_ for central Vienna Basin core samples of this study. The red line was fitted to the *Φ*_He_ data (*R*^2^ = 0.90) using the program BasinVis 2.0 introduced by Lee et al. (2020). Shale low-end, shale mean curve and shale high-end curve are modified from Kim et al. (2018) and represent a broad variety of mudstone compaction trends. Other displayed compaction trends are from the North Alpine Foreland Basin (Drews et al. 2018) and a compilation of offshore mudstone samples (Ewy et al. 2020). The data of this study plots systematically at lower porosities as expected for this depth range. The line Drews et al. (2018) from the North Alpine Foreland Basin plots close to the measured data of this study. Data from Ewy et al. (2020) plots close to the mean curve

Comparing the measured *Φ*_MICP_ and *Φ*_He_ depth trends of this study to previously published measured and calculated compaction-depth trends from different regions globally (e.g. Sclater and Christie 1980; Baldwin et al. 1985; Giles et al. 1998; Worden and Burley 2003; Mondol et al. 2007; Ewy et al. 2020; Nhabanga and Ringrose 2022) it seems that the Vienna Basin mudstones are shifted to systematically lower porosity values (Fig. 12). A slight porosity reduction may be due to drying alteration (“capillary suction”; Tang et al. 2011) and secondary carbonate cementation may be a further reason for variability beyond mechanical compaction effects in case of few individual samples (see above; Fig. 9). However, as a parallel shift in the general depth trend is visible for the whole sample set, we propose a regional uplift influence. According to the trend shift, regional uplift causing erosion of up to ~ 500 m of upper Miocene strata may be inferred. This estimate agrees well with erosion of 400–500 m, postulated recently by Harzhauser et al. (2022) based on the absence of uppermost Pannonian sediments (Gbely Fm.) in large parts of the Vienna Basin. According to these authors, erosion occurred distinctly after 8 Ma and is related to the basin inversion discussed by Peresson and Decker (1997a, b).

For the Bavarian part of the North Alpine Foreland Basin (~ 350 km west of the study area), uplift of about 500 m is proposed (Baran et al. 2014). A porosity depth trend of this basin plots close to data obtained in this study (Fig. 12). This supports the suggested similar regional uplift in the Vienna Basin.

The uplift of the basin also influences seal quality. One implication could be that capillary sealing properties in the Vienna Basin are beneficially shifted to shallower depths (e.g., properties of a 1000 m paleo-buried mudstone are observed already at 500 m present-day depth). Another possibility could be that the consolidated and, therefore, more brittle seals lose sealing capacity through fracturing during the uplift process (Ingram and Urai 1999). These implications should be kept in mind for future seal studies as additional factors influencing seal capacity despite capillary sealing properties.

## Conclusions

This study uses porosity-depth relationships to establish normal compaction trends for the Vienna Basin, based on which conclusions on the top seal quality distribution in the Badenian, Sarmatian, and Pannonian units in the central Vienna Basin are drawn. The porosity trends indicate that previously established mathematical mudstone compaction models are generally viable and that compaction in the central Vienna Basin follows a regional trend. From the comparison of the Vienna Basin data with global normal mudstone compaction trends, a possible regional uplift causing erosion of up to ~ 500 m of upper Miocene strata is inferred. Seal capacity models derived from measured capillary displacement pressures also follow a depth trend and agree with model-based hydrocarbon column height estimations, although the theoretical models lead to HCH values of > 1500 m for samples at burial depths of > 2 km, which are unlikely to occur in natural systems. These models would need calibration with actual field data; however, no true column height data are available for the complex and stacked Vienna Basin reservoirs. Furthermore, column heights in the Vienna Basin are likely not purely top seal-controlled in the majority of cases were the sampled intervals directly overly major reservoir sections. Nevertheless, the general conclusion that the seal capacity of the middle Miocene mudstone layers follows a relatively simple and basin-wide uniform depth trend seems to be valid based on the extensive petrophysical data set. The great theoretical HCH values already at moderate depths imply that capillary seal failure is rather unlikely despite the relatively high silt content of most of the investigated mudstone intervals. The results do not point to a significant influence of bulk mineralogy or silt contents on top seal capacity, although clay contents could not be determined due to the limited availability of sample material.

Although capillary seal integrity assuming water-wet seals is proven by the MICP data, we found geochemical evidence for hydrocarbon staining, presumably derived from vertically migrating oil. This correlates with the calculated column heights, indicating more staining in shallow and less consolidated samples vs. deeper samples with smaller mean displacement pore throat radii. This shows that while most investigated seals can be considered working top seals, a certain amount of fluid displacement into the seal may occur over geological time scales. Considering the potential impact of induced diagenesis e.g., due to CO_2_-enriched fluids in secondary storage scenarios, further investigations on the rates of vertical fluid migration, as well as potential rock-fluid interactions, are recommended. Also, vertical migration pathways besides purely fault-controlled scenarios may have to be considered in future hydrocarbon exploration of the Vienna Basin.

## Supplementary Information

Below is the link to the electronic supplementary material.

Supplementary file1 (XLSX 53 KB)

## Data Availability

All data supporting the findings of this study are available within the tables of the supplementary material files. In case raw data files are needed in another format, they are available from the corresponding author upon reasonable request.
